# Hand-foot syndrome in cancer patients on capecitabine: examining prevalence, impacts, and associated risk factors at a cancer centre in Malaysia

**DOI:** 10.1007/s00520-024-08490-7

**Published:** 2024-05-14

**Authors:** Teck Long King, Pei Jye Voon, Kah Hay Yuen, Dzul Azri Mohamed Noor

**Affiliations:** 1 Clinical Research Centre, Sarawak General Hospital, Ministry of Health, Kuching, Sarawak Malaysia; 2 School of Pharmaceutical Sciences, Universiti Sains Malaysia, Penang, Malaysia; 3 Department of Radiotherapy, Oncology and Palliative Care, Sarawak General Hospital, Ministry of Health, Kuching, Sarawak Malaysia

**Keywords:** Hand-foot syndrome, palmar-plantar erythrodysesthesia, capecitabine, fluoropyrimidines, chemotherapy

## Abstract

**Introduction:**

Hand-foot syndrome (HFS) significantly impacts quality of life in cancer patients undergoing capecitabine treatment. This study assessed capecitabine-associated HFS prevalence, its impacts on chemotherapy treatment, and identified risk factors in multiracial Malaysian patients.

**Methods:**

We included adult cancer patients receiving capecitabine at Sarawak General Hospital for at least two cycles from April 1, 2021 to June 30, 2022. HFS rates, time to HFS, and proportions of HFS-related treatment modifications were determined. Characteristics between patients with and without HFS were compared and multivariable logistic regression was used to identify risk factors for all-grade HFS and grade ≥2.

**Results:**

Among 369 patients, 185 (50.1%) developed HFS, with 14.6% experiencing grade ≥2 and 21.6% (40/185) underwent treatment modifications. Risk factors for all-grade HFS include older age (OR 1.03 95%CI 1.01, 1.06), prior chemotherapy (OR 2.09 95%CI 1.22, 3.58), higher capecitabine dose (OR 2.96 95%CI 1.62, 5.38), prolonged treatment (OR 1.36 95%CI 1.21, 1.51), folic acid intake (OR 3.27 95%CI 1.45, 7.35) and lower neutrophil count (OR 0.77 95%CI 0.66, 0.89). For HFS grade ≥2, older age (OR 1.04 95%CI 1.01, 1.08), female sex (OR 2.10 95%CI 1.05, 4.18), Chinese race (OR 2.10 95%CI 1.06, 4.18), and higher capecitabine dose (OR 2.62 95%CI 1.28, 5.35) are significant risk factors. Use of calcium channel blockers were associated with reduced risks of all-grade HFS (OR 0.27, 95%CI 0.12, 0.60) and grade ≥2 (OR 0.21 95%CI 0.06, 0.78).

**Conclusion:**

This study provides real-world data on capecitabine-induced HFS in Malaysian patients and identifies risk factors that may offer insights into its understanding and management.

**Supplementary Information:**

The online version contains supplementary material available at 10.1007/s00520-024-08490-7.

## Introduction

Capecitabine, as the prodrug of 5-fluorouracil (5-FU), is used either as monotherapy or in combination with other agents in adjuvant or palliative treatment for cancers. Although oral capecitabine has comparatively reduced gastrointestinal and bone marrow toxicities than intravenous fluorouracil [1], it is associated with a higher rate of hand-foot syndrome (HFS) [2].

HFS, also known as palmar-plantar erythrodysesthesia, is a well-documented and common adverse cutaneous reaction associated with chemotherapy. HFS is characterised by a range of symptoms including erythema, dysesthesia, pain, skin cracking, desquamation, and ulceration, primarily affecting the palms and soles of cancer patients undergoing capecitabine-based chemotherapy [3, 4]. HFS is typically graded using National Cancer Institute Common Terminology Criteria for Adverse Events (NCI-CTCAE, v5.0) grading scale [5]. While not posing a life-threatening risk, HFS can inflict pain and debilitation, disrupting patients' daily activities [6] and affecting their overall quality of life [7]. It may also lead to treatment interruptions and dose reductions [8, 9]. A study showed that 17-24% of metastatic colorectal cancer patients taking capecitabine experienced treatment modifications due to HFS [9].

A pooled analysis of clinical trials reported that up to 63% of the breast cancer patients taking capecitabine experienced HFS as an adverse event [10]. In a recent meta-analysis of clinical trials and observational studies, 68.3% of colorectal cancer patients experienced HFS after treatment with capecitabine alone, and 55.8% had HFS when treated with a combination of capecitabine and oxaliplatin. Moreover, 6% of them developed severe HFS. [11]. The study also highlighted disparities in HFS prevalence across different countries, with China having the highest rate at 55.1%, followed by Japan, Korea, and Italy [11]. However, to our knowledge, no study has reported the HFS prevalence in the Malaysian population.

There is a lack of real-world data on capecitabine-associated HFS and its impacts among the Malaysian patients. Sarawak General Hospital, as the largest hospital in Sarawak region, Malaysia, serves a population of around 2.5 million people. It is also providing cares to 80 to 90 per cent of cancer patients in Sarawak [12]. In this study, we aimed to evaluate the prevalence of HFS in cancer patients receiving capecitabine treatment at SGH, including the time to HFS onset and worsening, as well as any dose-reductions and treatment interruptions due to HFS. We also sought to explore the underlying demographic, clinical characteristics, and laboratory parameters as potential risk factors associated with the development of HFS associated with capecitabine treatment.

## Methods

In this retrospective, cross-sectional study, we included adult cancer patients aged 18 years and above who were receiving cancer care at Sarawak General Hospital and collected their capecitabine prescription from the hospital pharmacy between April 1, 2021, and June 30, 2022. We included patients who had received capecitabine monotherapy or combination therapy with another agent for a minimum of two treatment cycles. To ensure sufficient capecitabine exposure and avoid underestimation of HFS rate, we excluded those who prematurely discontinued their treatment (≤1 cycle) or lacked records indicating treatment continuation in their medical notes.

Upon initiation of capecitabine treatment, patients at our centre received urea cream as standard care for preventing HFS. Besides that, pharmacist counselling was given on non-pharmacological preventive measures, including avoiding exposure to extreme temperatures (e.g., washing hands with hot water) and minimising friction, trauma, and pressure on palms and soles [13]. In cases where HFS worsened to grade 2 or 3, capecitabine treatment might be interrupted and/or the dose reduced.

We retrieved information related to the patients' demographics, anthropometric measurements, comorbidities, concomitant medications, chemotherapy regimens, primary cancer site, cancer staging, Eastern Cooperative Oncology Group (ECOG) performance status, and their laboratory investigations at baseline before the first cycle of capecitabine treatment from their medical notes. We extracted the results of full blood counts, liver and renal function tests from the laboratory reports. Creatinine clearance was calculated using the Cockcroft-Gault method: ([140 – age, year] × [weight, kg]) x [0.85 if female]) / (72 x [serum creatinine, μmol/L] x 0.0113 [conversion factor to mg/dl]) [14]. The degree of HFS was assessed by attending doctors upon completion of every capecitabine treatment cycle and graded from 1 to 3 using the NCI-CTCAE, version 5 [5].

In our analysis, we calculated the HFS prevalence among the cancer patients receiving capecitabine, the median number of treatment cycles until the onset of HFS or worsened HFS of grade ≥2, and the proportions of HFS-related treatment interruptions and dose reductions. We collected baseline demographic and clinical characteristics, along with laboratory results, and compared between patients who developed HFS and those who did not. We conducted multivariable logistic regression analysis to explore the associations of demographic and clinical characteristics, with the occurrence of HFS of all-grade or grade ≥2. We employed another multivariable regression model for baseline laboratory findings, with age, sex, race, and prior chemotherapy history added as potential confounders to explore laboratory risk factors associated with the occurrence or worsening of HFS. We included both clinically and statistically significant variables (variables with *p*<0.10 from the univariable analysis were included) in the multivariable models. We used *jamovi* software, version 2.3 (The *jamovi* project, Sydney, Australia) and SPSS Statistics for Windows, version 16 (SPSS Inc., Chicago, Ill., USA) to perform our analyses. We set a *p*-value less than 0.05 as the statistical significance level.

However, we excluded breast cancer, cancer treatment intent, and capecitabine regime (monotherapy or combination therapy) from the multivariable analyses due to their high correlation with patient’s sex (breast cancer in females), cancer staging (palliative treatment intent in patients who had cancer stage IV), and capecitabine dose (patients receive lower capecitabine dose [2000mg/m^2^/day] when it is given in combination therapy), respectively. In addition, we also opted to include neutrophil count instead of total white blood cell count due to the high correlation between these two measures.

## Results

We included a total of 369 patients receiving capecitabine for analysis. The majority were female (50.4%), Chinese (37.7%), with a mean age of 57.0 years (standard deviation 11.7). Nearly two-thirds (65.9%) of the patients had colorectal cancer, followed by breast (16.5%) and gastric cancers (7.9%). Most of them were at stage III (42.2%) and IV (44.7%) and had an ECOG performance status of ≤1 (84.3%). Around 48.0% of the patients were taking capecitabine with palliative treatment intent. Slightly more than half (54.2%) were taking capecitabine in combination regimen, with majority (49.1%) receiving capecitabine in combination with oxaliplatin. Most patients were following the dosage regimen of 2000mg/m^2^/day (53.9%) or less (16.3%), with a median treatment duration of six cycles, as of the time of data collection. In terms of comorbidities, the majority had hypertension (44.4%) and dyslipidaemia (24.7%) and were taking calcium channel blockers (CCB) (16.8%) and statins (12.7%) (Table 1).

**Table 1:** Baseline demographics and clinical characteristics

Variables	Total *n* = 369	HFS *n* = 185	No HFS *n* = 184	*p*-value
Age (years), mean (SD)	57.0 (11.7)	57.8 (11.4)	56.1 (11.9)	0.164
Female sex, No. (%)	186 (50.4)	104 (56.2)	82 (44.6)	**0.025**
Race, No. (%)				**0.038**
Chinese	139 (37.7)	78 (42.2)	61 (33.2)	
Malays	94 (25.5)	49 (26.5)	45 (24.5)	
Iban	77 (20.9)	30 (16.2)	47 (25.5)	
Bidayuh	41 (11.1)	23 (12.4)	18 (9.8)	
Others	18 (4.9)	5 (2.7)	13 (7.1)	
Body weight (kg), mean (SD)	58.7 (12.6)	58.3 (12.1)	59.2 (13.2)	0.468
Body surface area (m^2^), mean (SD)	1.6 (0.2)	1.6 (0.2)	1.6 (0.2)	0.335
Primary cancer, No. (%)				**<0.001**
Colorectal	243 (65.9)	106 (57.3)	137 (74.5)	
Breast	61 (16.5)	46 (24.9)	15 (8.2)	
Gastric	29 (7.9)	12 (6.5)	17 (9.2)	
Nasopharygeal	13 (3.5)	10 (5.4)	3 (1.6)	
Pancreatic	9 (2.4)	4 (2.2)	5 (2.7)	
Oesophageal	5 (1.4)	3 (1.6)	2 (1.1)	
Others	9 (2.4)	4 (2.2)	5 (2.7)	
Cancer staging when capecitabine was prescribed, No. (%)				**0.001**
I	3 (0.8)	0 (0)	3 (1.7)	
II	44 (12.2)	25 (13.6)	19 (10.8)	
III	152 (42.2)	62 (33.7)	90 (51.1)	
IV	161 (44.7)	97 (52.7)	64 (36.4)	
ECOG, No. (%)				0.547
0	53 (18.9)	23 (17.0)	30 (20.5)	
1	184 (65.5)	88 (65.2)	96 (65.8)	
≥ 2	44 (15.6)	24 (17.8)	20 (13.7)	
Prior chemotherapy, No. (%)	172 (46.7)	109 (59.2)	63 (34.2)	**<0.001**
No. of previous regimen received, median (IQR)	0 (2)	1 (3)	0 (1)	**<0.001**
Capecitabine treatment intent, No. (%)				**0.001**
Neo-adjuvant/adjuvant	192 (52.0)	81 (43.8)	111 (60.3)	
Palliative	177 (48.0)	104 (56.2)	73 (39.7)	
Capecitabine treatment regimen, No. (%)				**<0.001**
Monotherapy	169 (45.8)	109 (58.9)	60 (32.6)	
Combination	200 (54.2)	76 (41.1)	124 (67.4)	
Oxaliplatin	181 (49.1)	64 (34.6)	117 (63.6)	
Gemcitabine	8 (2.2)	4 (2.2)	4 (2.2)	
Trastuzumab	6 (1.6)	4 (2.2)	2 (1.1)	
Bevacizumab	2 (0.5)	2 (1.1)	2 (0.5)	
Capecitabine dose prescribed (mg/m^2^/day), No. (%)				**<0.001**
<2000	60 (16.3)	23 (12.4)	37 (20.1)	
2000	199 (53.9)	80 (43.2)	119 (64.7)	
2500	110 (29.8)	82 (44.3)	28 (15.2)	
No. of capecitabine treatment cycles received, median (IQR)	6 (4)	8 (7)	5 (4)	**<0.001**
Comorbidities, No. (%)				
No known medical illness	139 (37.7)	74 (40.0)	65 (35.3)	0.354
Hypertension	164 (44.4)	75 (40.5)	89 (48.4)	0.130
Dyslipidaemia	91 (24.7)	43 (23.2)	48 (26.1)	0.526
Diabetes	73 (19.8)	32 (17.3)	41 (22.3)	0.229
Chronic kidney disease	2 (0.5)	0 (0)	2 (1.1)	0.155
Medication history, No. (%)				
Calcium channel blockers	62 (16.8)	20 (10.8)	42 (22.8)	**0.002**
Statins	47 (12.7)	19 (10.3)	28 (15.2)	0.154
Biguanides	42 (11.4)	17 (9.2)	25 (13.6)	0.184
RAS inhibitors				
ACEi	23 (6.2)	7 (3.8)	16 (8.7)	0.051
ARB	12 (3.3)	8 (4.3)	4 (2.2)	0.244
Beta blockers	22 (6.0)	9 (4.9)	13 (7.1)	0.372
Sulphonylureas	18 (4.9)	9 (4.9)	9 (4.9)	0.991
Folic acid	39 (10.6)	26 (14.1)	13 (7.1)	**0.029**
Vitamin B complex^a^	38 (10.3)	23 (12.4)	15 (8.2)	0.176

Bold values denote statistical significance (*p *< 0.05)

*SD* standard deviation, *IQR* interquartile range, *ECOG* Eastern Cooperative Oncology Group, *RAS* renin-angiotensin system, *ACEI* angiotensin-converting enzyme inhibitor, *ARB* angiotensin II receptor inhibitor

^a^ Vitamin B complex tablets contain thiamine, riboflavin, pyridoxine, cyanocobalamin, and nicotinamide

About half (50.1%) of the patients receiving capecitabine experienced HFS of all-grade, with 14.6% experiencing HFS of grade ≥2. The median number of treatment cycles until the onset of HFS was three cycles, and until HFS of grade ≥2 was five cycles. Among those who had HFS (n=185), about 22% had their chemotherapy treatment affected; 37 (20.0%) experienced treatment interruption, and 22 (11.9%) had their dosage reduced (Table 1).

**Table 2:** Capecitabine-associated hand-foot syndrome and treatment-related outcomes

Variables	Findings
Total HFS, No. (%)	185/369 (50.1)
Highest grade of HFS, No. (%)	
Grade 1	132/369 (35.8)
Grade 2	30/369 (8.1)
Grade 3	24/369 (6.5)
No. of cycles to HFS onset, median (IQR)	3 (3)
No. of cycles to HFS grade ≥2, median (IQR)	5 (3)
HFS-associated treatment interruption, No. (%)	37/185 (20.0)
HFS-associated dose reduction, No. (%)	22/185 (11.9)
HFS-associated treatment modification, No. (%)	40/185 (21.6)

*IQR* interquartile range

When comparing patients with HFS to those without the condition, we noted that the patients who had HFS were significantly more likely to be female and Chinese but less likely to be of Iban descent. Additionally, compared to patients who did not develop HFS, those with HFS had a higher proportion of patients diagnosed with breast cancer but had a smaller percentage of patients with colorectal cancer. Furthermore, individuals with HFS were more likely to be at stage IV of their cancer and had a history of prior chemotherapy. They were also more likely to be undergoing palliative chemotherapy, receiving capecitabine monotherapy, prescribed at a higher dose of 2500mg/m^2^/day, and have taken a significantly longer duration of chemotherapy. Besides that, we noted that individuals who had HFS were significantly less likely to be taking CCB and more likely to be taking supplementary folic acid (Table 1). In terms of laboratory parameters, capecitabine patients who had HFS had significantly lower counts of total white blood cells, specifically neutrophils, red blood cells, and platelets, along with a reduced globulin level at baseline, in comparison to those who did not develop HFS (Table 3).

**Table 3:** Baseline laboratory investigations

Variables	Unit	Total *n* = 369	HFS *n* = 185	No HFS *n* = 184	*p*-value
Full blood count					
Total white blood cell	10^3^/μL	7.6 (3.3)	7.1 (3.6)	8.1 (3.0)	**0.004**
Lymphocyte	10^3^/μL	2.0 (1.3)	2.0 (1.4)	2.1 (1.1)	0.767
Neutrophil	10^3^/μL	4.4 (2.2)	3.9 (1.7)	5.0 (2.5)	**<0.001**
Monocyte	10^3^/μL	0.6 (0.4)	0.6 (0.3)	0.7 (0.6)	0.114
Eosinophil	10^3^/μL	0.3 (0.3)	0.3 (0.3)	0.3 (0.2)	0.812
Basophil	10^3^/μL	0.05 (0.07)	0.04 (0.05)	0.05 (0.08)	0.525
Red blood cell	10^6^/μL	4.4 (0.8)	4.3 (0.8)	4.5 (0.9)	**0.046**
Platelet	10^3^/μL	318.0 (122.0)	294.9 (112.5)	341.3 (127.0)	**<0.001**
Renal profile					
Serum creatinine	μmol/L	73.2 (20.2)	71.8 (17.4)	74.6 (22.7)	0.175
Creatinine clearance	ml/min	80.0 (27.5)	78.3 (24.8)	81.6 (30.0)	0.255
Urea	mmol/L	4.0 (1.6)	4.0 (1.5)	4.1 (1.7)	0.700
Hepatic profile					
ALT	U/L	23.8 (18.6)	22.1 (14.1)	25.5 (22.1)	0.080
AST	U/L	29.6 (21.7)	28.2 (14.2)	31 (27.3)	0.265
ALP	U/L	97.5 (51.7)	95.4 (51.1)	99.6 (52.4)	0.445
Total bilirubin	μmol/L	8.3 (5.0)	8.2 (4.7)	8.4 (5.2)	0.797
Direct bilirubin	μmol/L	3.1 (2.9)	3.1 (1.9)	3.2 (3.6)	0.798
Albumin	g/L	40.7 (4.3)	40.9 (3.8)	40.4 (4.7)	0.292
Globulin	g/L	34.2 (5.9)	33.4 (5.8)	35.0 (5.9)	**0.009**

Bold values denote statistical significance (*p* < 0.05)

Data are presented as mean (standard deviation). *ALT* alanine aminotransferase, *AST* aspartate aminotransferase, *ALP* alkaline phosphatase

In the multivariable logistic regression analysis, we found that older age (OR 1.03, 95%CI 1.01, 1.06), prior treatment of chemotherapy (OR 2.09, 95% CI 1.22, 3.58), a higher capecitabine dose (OR 2.96, 95% CI 1.62, 5.38), and a longer treatment duration in cycles (OR 1.36, 95% CI 1.21, 1.51) were significantly associated with the occurrence of HFS in capecitabine patients. Our results also showed supplementary folic acid intake was significantly associated with higher risk of all-grade HFS (OR 3.27 95%CI 1.45, 7.35). Conversely, the concurrent use of CCB (OR 0.27, 95% CI 0.12, 0.60) was significantly associated with a reduced likelihood of developing HFS (Table 4). For HFS of grade ≥2, risk factors that showed significant associations were older age (OR 1.04, 95%CI 1.01, 1.08), female sex (OR 2.10, 95%CI 1.05, 4.18), Chinese race (OR 2.10, 95%CI 1.06 to 4.18), a higher capecitabine dose (OR 2.62, 95%CI 1.28, 5.35), and the absence of CCB in concomitant medications (OR 0.21, 95%CI 0.06, 0.78) (Table 4). In terms of laboratory investigations, only lower neutrophil count (OR 0.77 95%CI 0.66, 0.89) showed a significant association with the development of all-grade HFS in the multivariable analysis. None of the laboratory tests showed a significant association with the occurrence of grade ≥2 HFS, after controlling for other variables (Table 5). The univariable analysis findings can be found in the supplementary tables (Supplementary materials, Table S1 and Table S2).

**Table 4:** Multivariable logistic regression analysis of demographic and clinical factors associated with hand-foot syndrome in patients receiving capecitabine

Variables	HFS				HFS Grade ≥2			
	Adj. OR	95% CI		p-value	Adj. OR	95% CI		p-value
		Lower	Upper			Lower	Upper	
Age, year	1.03	1.01	1.06	**0.005**	1.04	1.01	1.08	**0.014**
Female sex	1.51	0.91	2.52	0.110	2.10	1.05	4.18	**0.035**
Chinese	1.15	0.67	1.98	0.609	2.10	1.06	4.18	**0.034**
Cancer stage IV	1.47	0.87	2.49	0.154	1.28	0.63	2.58	0.498
Prior chemotherapy	2.09	1.22	3.58	**0.008**	1.89	0.88	4.05	0.103
Capecitabine dose prescribed, 2500mg/m^2^/day	2.96	1.62	5.38	**<0.001**	2.62	1.28	5.35	**0.008**
Capecitabine treatment duration, cycle	1.36	1.21	1.51	**<0.001**	1.02	0.99	1.05	0.278
Calcium channel blockers	0.27	0.12	0.60	**0.001**	0.21	0.06	0.78	**0.019**
ACEi	1.00	0.32	3.15	1.000	1.06	0.19	5.80	0.951
Folic acid	3.27	1.45	7.35	**0.004**	1.30	0.47	3.61	0.612

Bold values denote statistical significance (*p  *< 0.05)

Adj. *OR* adjusted odd ratio, *CI* confidence interval, *ECOG* Eastern Cooperative Oncology Group, *IQR* interquartile range, *ACEI* angiotensin-converting enzyme inhibitor

**Table 5:** Multivariable logistic regression analysis of baseline laboratory findings associated with hand-foot syndrome in patients receiving capecitabine

Variables	HFS				HFS Grade ≥2			
	Adj. OR	95% CI		p-value	Adj. OR	95% CI		p-value
		Lower	Upper			Lower	Upper	
Age, year	1.02	1.00	1.04	0.110	1.02	0.99	1.07	0.114
Female sex	1.57	0.96	2.57	0.073	1.81	0.87	3.76	0.110
Chinese	1.29	0.75	2.21	0.361	2.21	1.02	4.80	**0.046**
Prior chemotherapy	2.56	1.52	4.32	**<0.001**	2.50	1.15	5.45	0.021
Neutrophil	0.77	0.66	0.89	**<0.001**	1.04	0.83	1.30	0.762
Red blood cell	0.83	0.58	1.19	0.307	0.78	0.43	1.40	0.400
Platelet, by 10 units	1.01	0.98	1.03	0.624	0.97	0.93	1.01	0.207
Globulin	1.00	0.96	1.05	0.862	1.01	0.94	1.09	0.825
ALT	0.99	0.98	1.01	0.242	-	-	-	-
ALP	-	-	-	-	0.99	0.98	1.00	0.094
Albumin	-	-	-	-	1.07	0.97	1.18	0.202
Eosinophil	-	-	-	-	0.39	0.07	2.17	0.280

Bold values denote statistical significance (*p *< 0.05)

Adj. *OR* adjusted odd ratio, *CI* confidence interval, *ALT* alanine aminotransferase, *ALP* alkaline phosphatase

## Discussion

The HFS prevalence in our study (all grades, 50.1%; grade ≥2, 14.6%) is consistent with previous findings in other populations [8, 10, 11]. Despite urea cream being routinely prescribed for HFS at our centre and pharmacist counselling given, we still observed around 30% of the HFS cases worsened, and 20% required capecitabine treatment adjustments. Randomised trial evaluating urea cream's efficacy for HFS has shown mixed results [15, 16]. Other potential treatments for HFS includes pyridoxine, celecoxib, and vitamin E [17–19], however, dose interruption or reduction remains the main approach to mitigate the condition [3, 4]. Future studies are warranted to determine treatment options to improve HFS management.

A higher capecitabine dose was significantly associated with an increased HFS rate in our study. Furthermore, HFS was more likely to occur in later capecitabine treatment cycles, consistent with previous studies demonstrating a higher occurrence with prolonged capecitabine treatment [20, 21]. HFS showed a dose-related and cumulative exposure association in patients receiving capecitabine in this study. Besides that, our univariable analysis suggested a lower HFS risk in the combination therapy group compared to those receiving capecitabine alone (Supplementary material, Table S1). However, as our patients received lower capecitabine dose when it is given in combination with other agents, the effect is confounded by the lower prescribed dose.

In addition, consistent with other studies [22, 23], our study showed a significant association between HFS and increasing age. Older patients often present with pre-existing comorbidities and are prescribed with multiple concurrent medications [22, 24]. Furthermore, a study showed that elderly patients may have reduced drug clearance, leading to higher capecitabine exposure compared to younger individuals [25]. These factors could contribute to the increased risk of HFS occurrence in older individuals.

We also found that female patients were more prone to develop HFS of grade ≥2. Sex difference in capecitabine toxicity has been previously reported [22, 26], possibly attributed to varying body compositions between males and females [27, 28]. A study showed that female colorectal cancer patients had more dose-limiting toxicity than male patients when given capecitabine based on their body surface area [26]. There is a growing call for sex-specific dosing strategies in cancer therapy [27, 29]. Future research could address this disparity between males and females, exploring alternative capecitabine dosing strategies beyond flat dose and body-surface-area-based dosing [27, 28].

Furthermore, studies have shown racial and ethnic differences in chemotherapy toxicities [30, 31]. In a study, African Americans and Hispanics demonstrated significantly higher capecitabine dose reductions due to side effects compared to non-Hispanic Caucasians, with African Americans also showing a non-significant increase in HFS risk relative to Caucasians [30]. Sarawak has a multiracial population comprising predominantly Ibans, Chinese, and Malays. Our findings indicate that Chinese patients had a significantly higher risk of grade ≥2 HFS compared to other racial groups. These disparities in HFS susceptibility may be attributed to genetic polymorphisms in capecitabine metabolism [32, 33], that leads to variation in toxicity profiles [34] across different races. However, a study in Singapore did not demonstrate such racial association with HFS risk, despite having a similar multiracial setting with a predominantly Chinese patient population (81%) [35]. Another potential explanation for the observed difference in HFS risk could be the racial variation in adherence to capecitabine treatment [36].

Our finding revealed a significant association of supplementary folic acid intake with increased HFS risk, consistent with a previous research by Yap *et al.* showing that folate levels are independent predictors of grade ≥2 HFS [35]. Besides that, an observational study in colorectal cancer patients found that use of folic acid supplements during capecitabine treatment significantly associated with increased risk of toxicities [37]. The presence of folate stablises the binding of the active fluorouracil metabolite of capecitabine, fluorodeoxyuridine monophosphate, to thymidylate synthase, forming a covalently bound ternary complex [38]. A higher level of exogenous folate may enhance the inhibition of thymidylate synthase, thereby contributing to toxicities [35].

Pyridoxine has been suggested to potentially prevent HFS [17, 39]. About 10% of our patients were prescribed vitamin B complex containing pyridoxine around the initiation of capecitabine treatment. However, we did not observe any effect on HFS in the present study.

Furthermore, the use of CCB was associated with a reduced risk of all-grade and grade ≥2 HFS in the present study, which was not previously reported. One potential explanation is that CCB may exhibit anti-inflammatory effects, as shown in *in-vitro* studies, which could mitigate HFS induced by capecitabine [40, 41]. While Kanbayashi *et al* found that concomitant use of renin-angiotensin system inhibitors was associated with a higher risk of HFS [42], we did not find such association in our study. The exact mechanism by which antihypertensive agents affects HFS is still unclear. In addition, others studies found that concomitant use of proton pump inhibitors may ameliorate capecitabine-induced HFS [43]; however, it is suggested that proton pump inhibitor use may reduce capecitabine bioavailability through increased gastric pH, potentially leading to decreased efficacy [44]. Future studies are needed to confirm the association between the use of CCB and HFS, understand the mechanisms on HFS, and explore possible interactions with capecitabine.

Besides that, we identified a negative association between baseline neutrophil count and the occurrence of HFS. While low baseline neutrophil counts and HFS have been associated with better outcomes after fluoropyrimidine-based chemotherapy, individually [45, 46], the relationship between neutrophil count and HFS remains unclear. This observation is counterintuitive, considering that HFS is thought to be related to inflammatory changes mediated by cyclooxygenase-2 [47]. However, the low baseline neutrophil level may be confounded by previous chemotherapy exposure, despite we had adjusted for this in the multivariable analysis. We noted that patients with prior chemotherapy treatment were more susceptible to HFS. Given the observational nature of our study, we cannot ascertain whether this is a delayed effect of previous chemotherapy. Furthermore, we did not gather information on the duration of previous treatment and the time gap to the initiation of capecitabine treatment.

This study has several limitations. Firstly, it was conducted in a single centre. However, Sarawak General Hospital serves the majority of cancer patients in Sarawak, making our study population representative of a large segment of cancer patients. Secondly, the retrospective design and use of secondary data from paper notes made our study prone to missing data and entry errors. Despite our efforts to capture capecitabine patients within the cross-sectional study period, we were unable to trace all patients' clinic documentations. Thirdly, we acknowledge the potential for inter-assessor variability in HFS assessment, despite the clear definitions provided in CTCAE version 5 [5]. The assessment of HFS was conducted by trained medical doctors in our centre.

In summary, our study provides real-world data on capecitabine-associated HFS among Malaysian patients. We also identified several risk factors associated with the occurrence and worsening of HFS with capecitabine use in cancer therapy. We showed that HFS is dose-limiting and associated with prolonged capecitabine exposure. Older age, Chinese race, and female sex were the significant demographic risk factors associated with HFS. We also found that folic acid intake may exacerbate the risk of HFS. Furthermore, our findings suggest a potential association between the use of CCB and a reduced risk of HFS and its worsening. We also observed a potential relationship between low neutrophil count and HFS. These findings enable us to assess the prevalence and impact of HFS in Malaysian patient population, while the identification of associated risk factors contributes to the understanding of HFS development that may inform strategies for its effective management.

### Supplementary information

ESM 1 (DOCX 24 kb)

## Data Availability

The data that support the findings of this study are available from the corresponding authors upon reasonable request.

## References

[1] Chintala L, Vaka S, Baranda J, Williamson SK (2011). Capecitabine versus 5-fluorouracil in colorectal cancer: where are we now?. Oncol Rev.

[2] Ter Veer E, Ngai LL, Valkenhoef GV, Mohammad NH, Anderegg MCJ, van Oijen MGH, van Laarhoven HWM (2017). Capecitabine, 5-fluorouracil and S-1 based regimens for previously untreated advanced oesophagogastric cancer: A network meta-analysis. Sci Rep.

[3] Kwakman JJM, Elshot YS, Punt CJA, Koopman M (2020). Management of cytotoxic chemotherapy-induced hand-foot syndrome. Oncol Rev.

[4] Gressett SM, Stanford BL, Hardwicke F (2006). Management of hand-foot syndrome induced by capecitabine. J Oncol Pharm Pract.

[5] U.S. Department of Health and Human Services (2017) Common Terminology Criteria for Adverse Events (CTCAE) Version 5.0. United States. Available from: https://ctep.cancer.gov/protocoldevelopment/electronic_applications/ctc.htm#ctc_50. Accessed 12 February 2024

[6] Hsu YH, Shen WC, Wang CH, Lin YF, Chen SC (2019). Hand-foot syndrome and its impact on daily activities in breast cancer patients receiving docetaxel-based chemotherapy. Eur J Oncol Nurs.

[7] Urakawa R, Tarutani M, Kubota K, Uejima E (2019). Hand Foot Syndrome Has the Strongest Impact on QOL in Skin Toxicities of Chemotherapy. J Cancer.

[8] Genetech USA (2015) XELODA (capecitabine) tablets, for oral use: Highlights for prescribing information. Available from: https://www.accessdata.fda.gov/drugsatfda_docs/label/2015/020896s037lbl.pdf. Accessed 31 October 2023

[9] Leicher LW, de Graaf JC, Coers W, Tascilar M, de Groot JW (2017). Tolerability of capecitabine monotherapy in metastatic colorectal cancer: a real-world study. Drugs R D.

[10] Blum JL, Barrios CH, Feldman N, Verma S, McKenna EF, Lee LF, Scotto N, Gralow J (2012). Pooled analysis of individual patient data from capecitabine monotherapy clinical trials in locally advanced or metastatic breast cancer. Breast Cancer Res Treat.

[11] Huang J, Ye S, Feng S, Zheng M, Zhong M (2023). Prevalence of hand-foot syndrome following chemotherapy for colorectal cancer: a systematic review and meta-analysis. Int J Colorectal Dis.

[12] CodeBlue (2021). MOH Mulls Building New Cancer Centre In Samarahan.

[13] Mohamad N, Othman NA, Yusup NL (2017). Cancer Drug Counselling: A Guide for Pharmacists.

[14] Cockcroft DW, Gault MH (1976). Prediction of creatinine clearance from serum creatinine. Nephron.

[15] Hofheinz RD, Gencer D, Schulz H, Stahl M, Hegewisch-Becker S, Loeffler LM, Kronawitter U, Bolz G, Potenberg J (2015). Mapisal Versus Urea Cream as Prophylaxis for Capecitabine-Associated Hand-Foot Syndrome: A Randomized Phase III Trial of the AIO Quality of Life Working Group. J Clin Oncol.

[16] Wolf SL, Qin R, Menon SP, Rowland KM, Thomas S, Delaune R, Christian D, Pajon ER, Satele DV (2010). Placebo-controlled trial to determine the effectiveness of a urea/lactic acid-based topical keratolytic agent for prevention of capecitabine-induced hand-foot syndrome: North Central Cancer Treatment Group Study N05C5. J Clin Oncol.

[17] Corrie PG, Bulusu R, Wilson CB, Armstrong G, Bond S, Hardy R, Lao-Sirieix S, Parashar D, Ahmad A (2012). A randomised study evaluating the use of pyridoxine to avoid capecitabine dose modifications. Br J Cancer.

[18] Zhang RX, Wu XJ, Wan DS, Lu ZH, Kong LH, Pan ZZ, Chen G (2012). Celecoxib can prevent capecitabine-related hand-foot syndrome in stage II and III colorectal cancer patients: result of a single-center, prospective randomized phase III trial. Ann Oncol.

[19] Yamamoto D, Yamamoto C, Iwase S, Kuroda Y, Odagiri H, Nagumo Y (2010). Efficacy of Vitamin E Treatment for Hand-Foot Syndrome in Patients Receiving Capecitabine. Breast Care (Basel).

[20] Stein A, Quidde J, Schroder JK, Gohler T, Tschechne B, Valdix AR, Hoffkes HG, Schirrmacher-Memmel S, Wohlfarth T (2016). Capecitabine in the routine first-line treatment of elderly patients with advanced colorectal cancer--results from a non-interventional observation study. BMC Cancer.

[21] Tanyi JL, Smith JA, Ramos L, Parker CL, Munsell MF, Wolf JK (2009). Predisposing risk factors for palmar-plantar erythrodysesthesia when using liposomal doxorubicin to treat recurrent ovarian cancer. Gynecol Oncol.

[22] Jiang Y, Mason M, Cho Y, Chittiprolu A, Zhang X, Harden K, Gong Y, Harris MR, Barton DL (2022). Tolerance to oral anticancer agent treatment in older adults with cancer: a secondary analysis of data from electronic health records and a pilot study of patient-reported outcomes. BMC Cancer.

[23] Meulendijks D, van Hasselt JGC, Huitema ADR, van Tinteren H, Deenen MJ, Beijnen JH, Cats A, Schellens JHM (2016). Renal function, body surface area, and age are associated with risk of early-onset fluoropyrimidine-associated toxicity in patients treated with capecitabine-based anticancer regimens in daily clinical care. Eur J Cancer.

[24] van Beek MWH, Roukens M, Jacobs WCH, Timmer-Bonte JNH, Kramers C (2018). Real-World Adverse Effects of Capecitabine Toxicity in an Elderly Population. Drugs Real World Outcomes.

[25] Louie SG, Ely B, Lenz HJ, Albain KS, Gotay C, Coleman D, Raghavan D, Shields AF, Gold PJ (2013). Higher capecitabine AUC in elderly patients with advanced colorectal cancer (SWOGS0030). Br J Cancer.

[26] Ilich AI, Danilak M, Kim CA, Mulder KE, Spratlin JL, Ghosh S, Chambers CR, Sawyer MB (2016). Effects of gender on capecitabine toxicity in colorectal cancer. J Oncol Pharm Pract.

[27] Ozdemir BC, Gerard CL, Espinosa da Silva C (2022) Sex and Gender Differences in Anticancer Treatment Toxicity: A Call for Revisiting Drug Dosing in Oncology. Endocrinology 163(6). 10.1210/endocr/bqac058 10.1210/endocr/bqac058 PMC9113364 35560216

[28] Wagner AD (2020). Sex differences in cancer chemotherapy effects, and why we need to reconsider BSA-based dosing of chemotherapy. ESMO Open.

[29] Ozdemir BC, Oertelt-Prigione S, Adjei AA, Borchmann S, Haanen JB, Letsch A, Mir O, Quaas A, Verhoeven RHA (2022). Investigation of sex and gender differences in oncology gains momentum: ESMO announces the launch of a Gender Medicine Task Force. Ann Oncol.

[30] Brazelton A, Yande S, Pope R, Johnson ML, Musher B, Trivedi MV (2022). Racial and ethnic differences in capecitabine toxicity in patients with gastrointestinal tract cancers. Ann Gastroenterol.

[31] Mizusawa J, Sato H, Rubinstein LV, Fujiwara T, Yonemori K, Hirakawa A (2023). Racial differences in longitudinal toxicities of anticancer agents in early phase cancer clinical trials. Cancer Med.

[32] Rosmarin D, Palles C, Church D, Domingo E, Jones A, Johnstone E, Wang H, Love S, Julier P (2014). Genetic markers of toxicity from capecitabine and other fluorouracil-based regimens: investigation in the QUASAR2 study, systematic review, and meta-analysis. J Clin Oncol.

[33] Lin S, Yue J, Guan X, Yuan P, Wang J, Luo Y, Fan Y, Cai R, Li Q (2019). Polymorphisms of MTHFR and TYMS predict capecitabine-induced hand-foot syndrome in patients with metastatic breast cancer. Cancer Commun (Lond).

[34] Ma Y, Tang L, Wang HX, Xu YC, Ma Y, Zhang FC (2012). Capecitabine for the treatment for advanced gastric cancer: efficacy, safety and ethnicity. J Clin Pharm Ther.

[35] Yap YS, Kwok LL, Syn N, Chay WY, Chia JWK, Tham CK, Wong NS, Lo SK, Dent RA (2017). Predictors of Hand-Foot Syndrome and Pyridoxine for Prevention of Capecitabine-Induced Hand-Foot Syndrome: A Randomized Clinical Trial. JAMA Oncol.

[36] Zahrina AK, Norsa'adah B, Hassan NB, Norazwany Y, Norhayati I, Roslan MH, Wan Nazuha WR (2014). Adherence to capecitabine treatment and contributing factors among cancer patients in Malaysia. Asian Pac J Cancer Prev.

[37] Kok DE, van Duijnhoven FJ, Lubberman FJ, McKay JA, Lanen AV, Winkels RM, Wesselink E, van Halteren HK, de Wilt JH (2024). Intake and biomarkers of folate and folic acid as determinants of chemotherapy-induced toxicities in patients with colorectal cancer: a cohort study. Am J Clin Nutr.

[38] Longley DB, Harkin DP, Johnston PG (2003). 5-fluorouracil: mechanisms of action and clinical strategies. Nat Rev Cancer.

[39] Lian S, Zhang X, Zhang Y, Zhao Q (2021). Pyridoxine for prevention of hand–foot syndrome caused by chemotherapy agents: a meta-analysis. Clin Exp Dermatol.

[40] Das R, Burke T, Van Wagoner DR, Plow EF (2009). L-type calcium channel blockers exert an antiinflammatory effect by suppressing expression of plasminogen receptors on macrophages. Circ Res.

[41] Shima E, Katsube M, Kato T, Kitagawa M, Hato F, Hino M, Takahashi T, Fujita H, Kitagawa S (2008). Calcium channel blockers suppress cytokine-induced activation of human neutrophils. Am J Hypertens.

[42] Kanbayashi Y, Taguchi T, Ishikawa T, Otsuji E, Takayama K (2023). Risk factors of capecitabine-induced hand-foot syndrome: a single-institution, retrospective study. Oncology.

[43] Takemura M, Ikemura K, Yoshinami T, Toyozumi Y, Shintani T, Ueda M, Shimazu K, Okuda M (2022). Proton pump inhibitors ameliorate capecitabine-induced hand-foot syndrome in patients with breast cancer: a retrospective study. Anticancer Res.

[44] Chu MP, Hecht JR, Slamon D, Wainberg ZA, Bang YJ, Hoff PM, Sobrero A, Qin S, Afenjar K (2017). association of proton pump inhibitors and capecitabine efficacy in advanced gastroesophageal cancer: secondary analysis of the TRIO-013/LOGiC randomized clinical trial. JAMA Oncol.

[45] Grothey A, Yoshino T, Bodoky G, Ciuleanu T, Garcia-Carbonero R, Garcia-Alfonso P, Van Cutsem E, Muro K, Mytelka DS (2018). Association of baseline absolute neutrophil counts and survival in patients with metastatic colorectal cancer treated with second-line antiangiogenic therapies: exploratory analyses of the RAISE trial and validation in an electronic medical record data set. ESMO Open.

[46] Zielinski C, Lang I, Beslija S, Kahan Z, Inbar MJ, Stemmer SM, Anghel R, Vrbanec D, Messinger D (2016). Predictive role of hand-foot syndrome in patients receiving first-line capecitabine plus bevacizumab for HER2-negative metastatic breast cancer. Br J Cancer.

[47] Lou Y, Wang Q, Zheng J, Hu H, Liu L, Hong D, Zeng S (2016). Possible Pathways of Capecitabine-Induced Hand-Foot Syndrome. Chem Res Toxicol.

[48] Subcommittee of Malaysian Guidelines for Good Clinical Practice (2018). Malaysian Guideline for Good Clinical Practice.

