# Supplementary material for: Hand-foot syndrome in cancer patients on capecitabine: examining prevalence, impacts, and associated risk factors at a cancer centre in Malaysia
Source: Support Care Cancer. 2024 May 14;32(6):345. doi: 10.1007/s00520-024-08490-7 (PMC11093791; doi:10.1007/s00520-024-08490-7)
Supplement: Supplementary file 1 — (DOCX 24 kb) [file 520_2024_8490_MOESM1_ESM.docx]

Table S1. Univariate logistic regression analysis of demographic factors and clinical characteristics associated with hand-foot syndrome in patients receiving capecitabine

|  | HFS | | | | HFS Grade ≥2 | | | |
| --- | --- | --- | --- | --- | --- | --- | --- | --- |
| Variables | OR | 95% CI | | *p*-value | OR | 95% CI | | *p*-value |
|  |  | Lower | Upper |  |  | Lower | Upper |  |
| Age | 1.01 | 1.00 | 1.03 | 0.165 | 1.03 | 1.00 | 1.06 | 0.037 |
| Female sex | 1.60 | 1.06 | 2.41 | 0.026 | 2.00 | 1.10 | 3.65 | 0.024 |
| Chinese | 1.47 | 0.962 | 2.25 | 0.075 | 2.36 | 1.32 | 4.24 | 0.004 |
| Body surface area (m^2)^ | 0.60 | 0.21 | 1.71 | 0.334 | 1.28 | 0.29 | 5.58 | 0.745 |
| Breast cancer | 3.73 | 2.00 | 6.96 | <0.001 | 3.16 | 1.65 | 6.06 | <0.001 |
| Cancer stage 4 | 1.95 | 1.28 | 2.98 | 0.002 | 1.92 | 1.06 | 3.47 | 0.031 |
| ECOG ≥ 1 | 1.26 | 0.69 | 2.30 | 0.453 | 1.11 | 0.46 | 2.67 | 0.812 |
| Prior chemotherapy | 2.79 | 1.83 | 4.26 | <0.001 | 3.19 | 1.71 | 5.96 | <0.001 |
| Capecitabine monotherapy | 2.96 | 1.94 | 4.54 | <0.001 | 3.70 | 1.96 | 6.99 | <0.001 |
| Capecitabine dose prescribed, 2500mg/m^2^/day | 4.48 | 2.77 | 7.25 | <0.001 | 3.81 | 2.10 | 6.92 | <0.001 |
| Capecitabine treatment duration, cycle | 1.38 | 1.26 | 1.51 | <0.001 | 1.03 | 1.00 | 1.06 | 0.035 |
| Calcium channel blockers | 0.41 | 0.23 | 0.73 | 0.002 | 0.26 | 0.08 | 0.85 | 0.026 |
| Statins | 0.64 | 0.34 | 1.19 | 0.156 | 0.51 | 0.17 | 1.47 | 0.211 |
| Biguanides | 0.64 | 0.34 | 1.24 | 0.186 | 0.97 | 0.39 | 2.42 | 0.946 |
| RAS inhibitors | 0.72 | 0.36 | 1.46 | 0.367 | 0.73 | 0.25 | 2.17 | 0.574 |
| ACEi | 0.41 | 0.17 | 1.03 | 0.058 | 0.54 | 0.12 | 2.37 | 0.412 |
| ARB | 2.03 | 0.60 | 6.88 | 0.253 | 1.17 | 0.25 | 5.51 | 0.840 |
| Beta-blockers | 0.67 | 0.28 | 1.61 | 0.375 | 0.92 | 0.26 | 3.21 | 0.891 |
| Sulphonyureas | 0.99 | 0.39 | 2.56 | 0.991 | 0.72 | 0.16 | 3.22 | 0.666 |
| Folic acid | 2.15 | 1.07 | 4.33 | 0.032 | 1.07 | 0.43 | 2.69 | 0.889 |
| Vitamin B complex^a^ | 1.60 | 0.81 | 3.17 | 0.179 | 0.66 | 0.23 | 1.95 | 0.452 |

OR, odd ratio; CI, confidence interval; ECOG, Eastern Cooperative Oncology Group; IQR, interquartile range; RAS, renin-angiotensin system; ACEI, angiotensin-converting enzyme inhibitor; ARB, angiotensin II receptor inhibitor.

^a^ Vitamin B complex tablets contain thiamine, riboflavin, pyridoxine, cyanocobalamin, and nicotinamide.

Table S2. Univariate logistic regression analysis of baseline laboratory investigations associated with hand-foot syndrome in patients receiving capecitabine

|  | HFS | | | | HFS Grade ≥2 | | | |
| --- | --- | --- | --- | --- | --- | --- | --- | --- |
| Variables | OR | 95% CI | | *p*-value | OR | 95% CI | | *p*-value |
|  |  | Lower | Upper |  |  | Lower | Upper |  |
| Total white blood cell | 0.90 | 0.84 | 0.97 | 0.006 | 0.85 | 0.75 | 0.97 | 0.013 |
| Lymphocyte | 0.98 | 0.82 | 1.16 | 0.767 | 0.89 | 0.66 | 1.19 | 0.428 |
| Neutrophil | 0.76 | 0.68 | 0.85 | <0.001 | 0.85 | 0.73 | 1.01 | 0.057 |
| Monocyte | 0.57 | 0.27 | 1.21 | 0.141 | 0.61 | 0.19 | 1.95 | 0.404 |
| Eosinophil | 1.10 | 0.51 | 2.35 | 0.811 | 0.17 | 0.03 | 0.936 | 0.042 |
| Basophil | 0.34 | 0.01 | 9.84 | 0.531 | 1.56 | 0.02 | 98.47 | 0.834 |
| Red blood cell | 0.76 | 0.58 | 1.00 | 0.053 | 0.65 | 0.42 | 1.01 | 0.057 |
| Platelet, by 10 unit | 0.97 | 0.95 | 0.99 | <0.001 | 0.95 | 0.92 | 0.98 | <0.001 |
| Serum creatinine | 0.99 | 0.98 | 1.00 | 0.178 | 1.00 | 0.99 | 1.01 | 0.968 |
| Creatinine clearance | 1.00 | 0.99 | 1.00 | 0.255 | 0.99 | 0.98 | 1.00 | 0.173 |
| Urea | 0.98 | 0.86 | 1.11 | 0.699 | 1.06 | 0.89 | 1.27 | 0.492 |
| ALT | 0.99 | 0.98 | 1.00 | 0.086 | 0.99 | 0.98 | 1.01 | 0.473 |
| AST | 0.99 | 0.98 | 1.00 | 0.273 | 1.00 | 0.99 | 1.01 | 0.935 |
| ALP | 1.00 | 0.99 | 1.00 | 0.445 | 0.99 | 0.98 | 1.00 | 0.021 |
| Total bilirubin | 0.99 | 0.95 | 1.04 | 0.796 | 0.98 | 0.92 | 1.04 | 0.512 |
| Direct bilirubin | 0.99 | 0.92 | 1.07 | 0.798 | 0.99 | 0.88 | 1.11 | 0.817 |
| Albumin | 1.03 | 0.98 | 1.08 | 0.292 | 1.08 | 1.00 | 1.17 | 0.045 |
| Globulin | 0.95 | 0.92 | 0.99 | 0.010 | 0.93 | 0.88 | 0.98 | 0.007 |

OR, odd ratio; CI, confidence interval; ALT, alanine transaminase; AST, aspartate transaminase; ALP, alkaline phosphatase
